# Bis(1,10-phenanthroline-κ^2^ *N*,*N*′)(sulfato-κ^2^ *O*,*O*′)zinc(II) propane-1,3-diol solvate

**DOI:** 10.1107/S1600536810014194

**Published:** 2010-04-24

**Authors:** Jiang-Dong Cui, Kai-Long Zhong, Yan-Yun Liu

**Affiliations:** a Department of Applied Chemistry, Nanjing College of Chemical Technology, Nanjing 210048, People’s Republic of China

## Abstract

In the title compound, [Zn(SO_4_)(C_12_H_8_N_2_)_2_]·C_3_H_8_O_2_, the Zn^2+^ ion (site symmetry 2) is coordinated by two chelating 1,10-phenanthroline ligands and an *O*,*O*′-bidentate sulfate ion (S site symmetry 2), resulting in a distorted *cis*-ZnO_2_N_4_ octa­hedral geometry for the metal ion. The complete propane-1,3-diol mol­ecule is generated by crystallographic twofold symmetry and two O—H⋯O hydrogen bonds are formed with the uncoordinated O atoms of the sulfate group.

## Related literature

For related structures and background references, see: Zhong (2010*a* ▶,*b* ▶). 
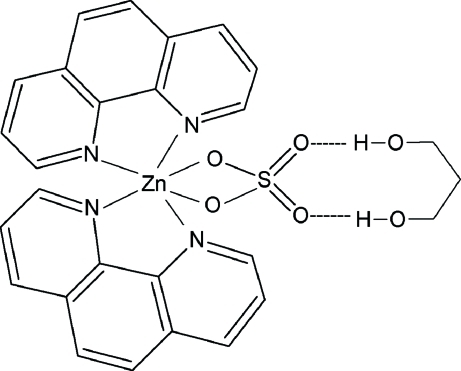


## Experimental

### 

#### Crystal data

[Zn(SO_4_)(C_12_H_8_N_2_)_2_]·C_3_H_8_O_2_
*M* *_r_* = 597.96
Monoclinic, 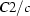

*a* = 18.330 (4) Å
*b* = 12.406 (3) Å
*c* = 13.215 (3) Å
β = 121.78 (3)°
*V* = 2554.6 (13) Å^3^
*Z* = 4
Mo *K*α radiation
μ = 1.10 mm^−1^
*T* = 223 K
0.25 × 0.20 × 0.12 mm

#### Data collection

Rigaku Mercury CCD diffractometer
Absorption correction: multi-scan (*REQAB*; Jacobson, 1998 ▶) *T*_min_ = 0.790, *T*_max_ = 1.000
7464 measured reflections
2241 independent reflections
1932 reflections with *I* > 2σ(*I*)
*R*_int_ = 0.039

#### Refinement

*R*[*F* ^2^ > 2σ(*F* ^2^)] = 0.044
*wR*(*F* ^2^) = 0.104
*S* = 1.08
2241 reflections
179 parameters
H-atom parameters constrained
Δρ_max_ = 0.63 e Å^−3^
Δρ_min_ = −0.34 e Å^−3^

### 

Data collection: *CrystalClear* (Rigaku, 2007 ▶); cell refinement: *CrystalClear*; data reduction: *CrystalClear*; program(s) used to solve structure: *SHELXS97* (Sheldrick, 2008 ▶); program(s) used to refine structure: *SHELXL97* (Sheldrick, 2008 ▶); molecular graphics: *XP* in *SHELXTL* (Sheldrick, 2008 ▶); software used to prepare material for publication: *SHELXTL*.

## Supplementary Material

Crystal structure: contains datablocks global, I. DOI: 10.1107/S1600536810014194/hb5408sup1.cif

Structure factors: contains datablocks I. DOI: 10.1107/S1600536810014194/hb5408Isup2.hkl

Additional supplementary materials:  crystallographic information; 3D view; checkCIF report

## Figures and Tables

**Table:** 

Zn1—N2	2.145 (3)
Zn1—N1	2.147 (3)
Zn1—O1	2.174 (2)

**Table:** 

N2—Zn1—N1	77.87 (10)
O1—Zn1—O1^i^	65.58 (11)

Symmetry code: (i) 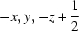.

**Table 2:** Hydrogen-bond geometry (Å, °)

*D*—H⋯*A*	*D*—H	H⋯*A*	*D*⋯*A*	*D*—H⋯*A*
O3—H3*B*⋯O2	0.82	1.95	2.727 (4)	157

**Fig. 1.**
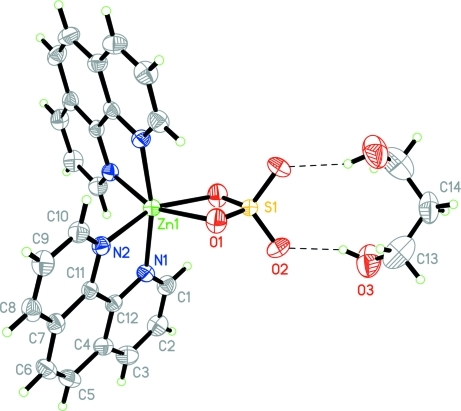
The molecular structure of (I) with displacement ellipsoids drawn at the 50% probability level. The dashed lines represent O—H···O interactions. Unlabeled atoms are related to the labelled atoms by the symmetry operator(-x, y, - z + 1/2).

## supplementary crystallographic information

### Comment

The title compound, (I), was obtained unitentionally during an attempt to synthesize coordination polymers of Zn(II) with 1,10-phenanthroline as second ligand *via* a solvothermal reaction.  It is isomorphous with the recently reported cobalt(II) structure (Zhong 2010a).

In this study, the structure of Zn^II^ complexe with bidentate-chelating sulfate ligand, *viz*. [ZnSO_4_(phen)_2_].C_3_H_8_O_2_, has been characterized. each Zn^II^ metal ion is six-coordinated in a distorted octahedral manner by four N atoms from two chelating phen ligands and two O atoms from a bidentate-chelating sulfate ligand. The formula unit lies on a twofold rotation axis [symmetry code: -*x*, *y*, - *z* + 1/2] passes through the Zn^II^ and S atoms, and also through the central carbon of the propane-1,3-diol solvent molecule, in C/2c . Around the twofold axis two planar phen ligands are arranged in a propeller manner. Intermolecular O—H···O hydrogen bonds help to further stabilize the crystal structure(see Fig. 1). Selected coordination bond distances and angles in Table 1 and intermolecular hydrogen bond see Table 2.

We discuss the title complexe and compare it with the previously reported compound [ZnSO_4_(C_10_H_8_N_2_)_2_].C_2_H_6_O_2_, (II) (C_10_H_8_N_2_ is 2,2'-bipyridine; Zhong, 2010b). In (I), the Zn^II^ metal ions has an octahedral coordinaiton environment is in good agreement with that observed in (II), The Zn—O bond distance [2.174 (2) Å] and the Zn—N bond distances [2.145 (3)-2.147 (3) Å] are close to those found in (II) [2.1811 (15)Å and 2.1287 (17)-2.1452 (17) Å; respectively]. The N—Zn—N angle [77.87 (10)°] and the O—Zn—O angle [65.58 (11)°] in (I) are also comparable with values reported in (II) [76.61 (7)° and 65.64 (8)° respectively], The dihedral angle (79.8°) between the two chelating NCCN groups is slightly less than that found in (II), 81.1 (1)°.

### Experimental

0.2 mmol phen, 0.1 mmol melamine, 0.1 mmol ZnO_4_.7H_2_O, 2.0 ml propane-1,3-diol and 1.0 ml water were mixed and placed in a thick Pyrex tube, which was sealed and heated to 413 K for 96 h.  After cooling, colorless blocks of (I) were obtained.

### Refinement

The H atoms of phen were positioned geometrically and allowed to ride on their parent atoms, with C—H = 0.93 Å and *U*_iso_(H) = 1.2*U*_eq_(C). The H atoms of central carbon of propane-1,3-diol were located in difference Fourier syntheses and were freely refined [C—H = 0.97 Å] and *U*_iso_(H) = 1.2*U*_eq_(C), whereas other H atoms were placed in geometrically idealized positions and refined as riding atoms, with C—H = 0.97 Å and O—H = 0.82 Å; *U*_iso_(H) = 1.2*U*_eq_(C) and 1.5*U*_eq_(O).

### Crystal data

**Table:**

[Zn(SO_4_)(C_12_H_8_N_2_)_2_]·C_3_H_8_O_2_	*F*(000) = 1232
*M**_r_* = 597.96	*D*_x_ = 1.555 Mg m^−^^3^
Monoclinic, *C*2/*c*	Mo *K*α radiation, λ = 0.71073 Å
Hall symbol: -C 2yc	Cell parameters from 4259 reflections
*a* = 18.330 (4) Å	θ = 3.2–27.5°
*b* = 12.406 (3) Å	µ = 1.10 mm^−^^1^
*c* = 13.215 (3) Å	*T* = 223 K
β = 121.78 (3)°	Block, colourless
*V* = 2554.6 (13) Å^3^	0.25 × 0.20 × 0.12 mm
*Z* = 4	

### Data collection

**Table:**

Rigaku Mercury CCD diffractometer	2241 independent reflections
Radiation source: fine-focus sealed tube	1932 reflections with *I* > 2σ(*I*)
Graphite Monochromator	*R*_int_ = 0.039
Detector resolution: 28.5714 pixels mm^-1^	θ_max_ = 25.0°, θ_min_ = 3.2°
ω scans	*h* = −21→15
Absorption correction: multi-scan (REQAB; Jacobson, 1998)	*k* = −14→13
*T*_min_ = 0.790, *T*_max_ = 1.000	*l* = −14→15
7464 measured reflections	

### Refinement

**Table:**

Refinement on *F*^2^	Secondary atom site location: difference Fourier map
Least-squares matrix: full	Hydrogen site location: inferred from neighbouring sites
*R*[*F*^2^ > 2σ(*F*^2^)] = 0.044	H-atom parameters constrained
*wR*(*F*^2^) = 0.104	*w* = 1/[σ^2^(*F*_o_^2^) + (0.0559*P*)^2^ + 1.7701*P*] where *P* = (*F*_o_^2^ + 2*F*_c_^2^)/3
*S* = 1.08	(Δ/σ)_max_ < 0.001
2241 reflections	Δρ_max_ = 0.63 e Å^−^^3^
179 parameters	Δρ_min_ = −0.34 e Å^−^^3^
0 restraints	Extinction correction: *SHELXL97* (Sheldrick, 2008), Fc^*^=kFc[1+0.001xFc^2^λ^3^/sin(2θ)]^-1/4^
Primary atom site location: structure-invariant direct methods	Extinction coefficient: 0.0021 (4)

### Special details

**Table:**

Geometry. All esds (except the esd in the dihedral angle between two l.s. planes) are estimated using the full covariance matrix. The cell esds are taken into account individually in the estimation of esds in distances, angles and torsion angles; correlations between esds in cell parameters are only used when they are defined by crystal symmetry. An approximate (isotropic) treatment of cell esds is used for estimating esds involving l.s. planes.
Refinement. Refinement of *F*^2^ against ALL reflections. The weighted *R*-factor wR and goodness of fit *S* are based on *F*^2^, conventional *R*-factors *R* are based on *F*, with *F* set to zero for negative *F*^2^. The threshold expression of *F*^2^ > σ(*F*^2^) is used only for calculating *R*-factors(gt) etc. and is not relevant to the choice of reflections for refinement. *R*-factors based on *F*^2^ are statistically about twice as large as those based on *F*, and *R*- factors based on ALL data will be even larger.

### Fractional atomic coordinates and isotropic or equivalent isotropic displacement parameters (Å^2^)

**Table:**

	*x*	*y*	*z*	*U*_iso_*/*U*_eq_	
Zn1	0.0000	0.31613 (4)	0.2500	0.0275 (2)	
S1	0.0000	0.53713 (8)	0.2500	0.0261 (3)	
O2	0.05644 (17)	0.6038 (2)	0.2306 (2)	0.0501 (7)	
O1	0.05155 (15)	0.46343 (17)	0.35278 (18)	0.0386 (6)	
N1	0.08295 (17)	0.29637 (19)	0.1822 (2)	0.0279 (6)	
N2	0.09735 (17)	0.21096 (19)	0.3797 (2)	0.0271 (6)	
C9	0.1742 (2)	0.1058 (3)	0.5575 (3)	0.0365 (8)	
H9A	0.1784	0.0812	0.6268	0.044*	
C5	0.2775 (2)	0.1280 (3)	0.2899 (3)	0.0354 (8)	
H5A	0.3165	0.1084	0.2684	0.042*	
C1	0.0755 (2)	0.3403 (3)	0.0849 (3)	0.0347 (8)	
H1A	0.0310	0.3887	0.0408	0.042*	
C7	0.2267 (2)	0.1149 (2)	0.4258 (2)	0.0287 (7)	
C2	0.1314 (2)	0.3164 (3)	0.0470 (3)	0.0370 (8)	
H2A	0.1238	0.3479	−0.0218	0.044*	
C4	0.2084 (2)	0.1990 (2)	0.2154 (3)	0.0302 (7)	
C3	0.1975 (2)	0.2465 (3)	0.1113 (3)	0.0374 (8)	
H3A	0.2353	0.2301	0.0867	0.045*	
C6	0.2869 (2)	0.0887 (3)	0.3919 (3)	0.0348 (8)	
H6A	0.3331	0.0440	0.4405	0.042*	
C8	0.2340 (2)	0.0767 (2)	0.5317 (3)	0.0336 (8)	
H8A	0.2794	0.0321	0.5831	0.040*	
C12	0.14832 (19)	0.2268 (2)	0.2462 (2)	0.0249 (6)	
C10	0.1065 (2)	0.1729 (3)	0.4796 (3)	0.0325 (7)	
H10A	0.0660	0.1918	0.4985	0.039*	
C11	0.15675 (19)	0.1826 (2)	0.3525 (2)	0.0246 (6)	
O3	0.0629 (3)	0.8156 (2)	0.1807 (3)	0.0736 (10)	
H3B	0.0462	0.7553	0.1845	0.110*	
C14	0.0000	0.9448 (4)	0.2500	0.0615 (17)	
C13	0.0766 (4)	0.8753 (4)	0.2756 (5)	0.0860 (17)	
H13A	0.1258	0.9217	0.3005	0.103*	
H13B	0.0905	0.8272	0.3412	0.103*	
H14B	−0.0159	0.9906	0.1817	0.103*	

### Atomic displacement parameters (Å^2^)

**Table:**

	*U*^11^	*U*^22^	*U*^33^	*U*^12^	*U*^13^	*U*^23^
Zn1	0.0250 (3)	0.0253 (3)	0.0328 (3)	0.000	0.0157 (2)	0.000
S1	0.0229 (6)	0.0234 (5)	0.0330 (6)	0.000	0.0153 (5)	0.000
O2	0.0474 (16)	0.0435 (14)	0.0763 (18)	−0.0092 (13)	0.0442 (14)	0.0034 (13)
O1	0.0360 (14)	0.0343 (12)	0.0306 (12)	0.0007 (11)	0.0073 (10)	0.0020 (9)
N1	0.0247 (14)	0.0276 (13)	0.0297 (13)	0.0026 (11)	0.0133 (11)	0.0027 (10)
N2	0.0284 (15)	0.0251 (13)	0.0289 (13)	−0.0018 (11)	0.0160 (11)	−0.0031 (10)
C9	0.045 (2)	0.0368 (18)	0.0274 (16)	0.0002 (16)	0.0188 (15)	0.0027 (13)
C5	0.0276 (18)	0.0409 (19)	0.0379 (17)	0.0037 (15)	0.0175 (14)	−0.0052 (14)
C1	0.0340 (19)	0.0337 (18)	0.0326 (17)	−0.0015 (15)	0.0150 (14)	0.0038 (13)
C7	0.0282 (17)	0.0241 (15)	0.0268 (15)	−0.0022 (14)	0.0096 (13)	−0.0039 (12)
C2	0.044 (2)	0.0414 (18)	0.0292 (16)	−0.0030 (17)	0.0218 (15)	0.0013 (14)
C4	0.0275 (17)	0.0332 (17)	0.0303 (16)	−0.0019 (14)	0.0155 (14)	−0.0057 (12)
C3	0.037 (2)	0.047 (2)	0.0347 (17)	−0.0002 (17)	0.0231 (15)	−0.0012 (15)
C6	0.0260 (17)	0.0337 (17)	0.0347 (17)	0.0062 (15)	0.0091 (14)	−0.0053 (13)
C8	0.0318 (18)	0.0299 (17)	0.0280 (16)	0.0039 (15)	0.0080 (14)	0.0048 (12)
C12	0.0239 (16)	0.0236 (14)	0.0245 (14)	−0.0060 (13)	0.0110 (12)	−0.0051 (12)
C10	0.0377 (19)	0.0332 (17)	0.0318 (16)	−0.0006 (15)	0.0219 (15)	−0.0003 (13)
C11	0.0258 (16)	0.0211 (14)	0.0254 (14)	−0.0021 (13)	0.0124 (12)	−0.0042 (11)
O3	0.122 (3)	0.0507 (17)	0.089 (2)	−0.0088 (18)	0.084 (2)	−0.0004 (16)
C14	0.092 (5)	0.032 (3)	0.070 (4)	0.000	0.049 (4)	0.000
C13	0.084 (4)	0.086 (4)	0.084 (3)	−0.031 (3)	0.041 (3)	−0.005 (3)

### Geometric parameters (Å, °)

**Table:**

Zn1—N2^i^	2.145 (3)	C1—H1A	0.9300
Zn1—N2	2.145 (3)	C7—C11	1.407 (4)
Zn1—N1	2.147 (3)	C7—C8	1.415 (4)
Zn1—N1^i^	2.147 (3)	C7—C6	1.429 (5)
Zn1—O1	2.174 (2)	C2—C3	1.363 (5)
Zn1—O1^i^	2.174 (2)	C2—H2A	0.9300
S1—O2	1.449 (2)	C4—C12	1.403 (5)
S1—O2^i^	1.449 (2)	C4—C3	1.410 (4)
S1—O1^i^	1.491 (2)	C3—H3A	0.9300
S1—O1	1.491 (2)	C6—H6A	0.9300
N1—C1	1.335 (4)	C8—H8A	0.9300
N1—C12	1.352 (4)	C12—C11	1.439 (4)
N2—C10	1.327 (4)	C10—H10A	0.9300
N2—C11	1.360 (4)	O3—C13	1.361 (6)
C9—C8	1.357 (5)	O3—H3B	0.8200
C9—C10	1.395 (5)	C14—C13^i^	1.527 (7)
C9—H9A	0.9300	C14—C13	1.527 (7)
C5—C6	1.356 (5)	C14—H14B	0.9728
C5—C4	1.427 (5)	C13—H13A	0.9700
C5—H5A	0.9300	C13—H13B	0.9700
C1—C2	1.391 (5)		
			
N2^i^—Zn1—N2	105.08 (13)	N1—C1—H1A	118.7
N2^i^—Zn1—N1	94.08 (10)	C2—C1—H1A	118.7
N2—Zn1—N1	77.87 (10)	C11—C7—C8	117.3 (3)
N2^i^—Zn1—N1^i^	77.87 (10)	C11—C7—C6	119.6 (3)
N2—Zn1—N1^i^	94.08 (10)	C8—C7—C6	123.1 (3)
N1—Zn1—N1^i^	166.89 (13)	C3—C2—C1	119.5 (3)
N2^i^—Zn1—O1	156.26 (9)	C3—C2—H2A	120.3
N2—Zn1—O1	96.15 (9)	C1—C2—H2A	120.3
N1—Zn1—O1	100.74 (9)	C12—C4—C3	116.7 (3)
N1^i^—Zn1—O1	90.32 (9)	C12—C4—C5	119.9 (3)
N2^i^—Zn1—O1^i^	96.15 (9)	C3—C4—C5	123.4 (3)
N2—Zn1—O1^i^	156.26 (9)	C2—C3—C4	119.9 (3)
N1—Zn1—O1^i^	90.32 (9)	C2—C3—H3A	120.1
N1^i^—Zn1—O1^i^	100.74 (9)	C4—C3—H3A	120.1
O1—Zn1—O1^i^	65.58 (11)	C5—C6—C7	121.0 (3)
O2—S1—O2^i^	110.4 (2)	C5—C6—H6A	119.5
O2—S1—O1^i^	110.96 (14)	C7—C6—H6A	119.5
O2^i^—S1—O1^i^	110.01 (15)	C9—C8—C7	119.4 (3)
O2—S1—O1	110.01 (14)	C9—C8—H8A	120.3
O2^i^—S1—O1	110.96 (14)	C7—C8—H8A	120.3
O1^i^—S1—O1	104.33 (18)	N1—C12—C4	123.2 (3)
O2—S1—Zn1	124.79 (11)	N1—C12—C11	117.2 (3)
O2^i^—S1—Zn1	124.79 (11)	C4—C12—C11	119.5 (3)
O1^i^—S1—Zn1	52.17 (9)	N2—C10—C9	123.0 (3)
O1—S1—Zn1	52.17 (9)	N2—C10—H10A	118.5
S1—O1—Zn1	95.04 (11)	C9—C10—H10A	118.5
C1—N1—C12	118.0 (3)	N2—C11—C7	122.6 (3)
C1—N1—Zn1	128.2 (2)	N2—C11—C12	118.1 (3)
C12—N1—Zn1	113.7 (2)	C7—C11—C12	119.3 (3)
C10—N2—C11	118.1 (3)	C13—O3—H3B	109.5
C10—N2—Zn1	128.9 (2)	C13^i^—C14—C13	111.2 (5)
C11—N2—Zn1	113.02 (19)	C13^i^—C14—H14B	109.5
C8—C9—C10	119.7 (3)	C13—C14—H14B	109.1
C8—C9—H9A	120.2	O3—C13—C14	113.7 (4)
C10—C9—H9A	120.2	O3—C13—H13A	108.8
C6—C5—C4	120.7 (3)	C14—C13—H13A	108.8
C6—C5—H5A	119.7	O3—C13—H13B	108.8
C4—C5—H5A	119.7	C14—C13—H13B	108.8
N1—C1—C2	122.6 (3)	H13A—C13—H13B	107.7
			
N2^i^—Zn1—S1—O2	−110.63 (16)	O1^i^—Zn1—N2—C10	114.9 (3)
N2—Zn1—S1—O2	69.37 (16)	S1—Zn1—N2—C10	87.6 (3)
N1—Zn1—S1—O2	−10.36 (14)	N2^i^—Zn1—N2—C11	88.39 (19)
N1^i^—Zn1—S1—O2	169.64 (14)	N1—Zn1—N2—C11	−2.61 (19)
O1—Zn1—S1—O2	89.31 (18)	N1^i^—Zn1—N2—C11	166.92 (19)
O1^i^—Zn1—S1—O2	−90.69 (18)	O1—Zn1—N2—C11	−102.3 (2)
N2^i^—Zn1—S1—O2^i^	69.37 (16)	O1^i^—Zn1—N2—C11	−64.3 (3)
N2—Zn1—S1—O2^i^	−110.63 (16)	S1—Zn1—N2—C11	−91.61 (19)
N1—Zn1—S1—O2^i^	169.64 (14)	C12—N1—C1—C2	0.6 (5)
N1^i^—Zn1—S1—O2^i^	−10.36 (14)	Zn1—N1—C1—C2	−176.5 (2)
O1—Zn1—S1—O2^i^	−90.69 (18)	N1—C1—C2—C3	−0.8 (5)
O1^i^—Zn1—S1—O2^i^	89.31 (18)	C6—C5—C4—C12	−1.4 (5)
N2^i^—Zn1—S1—O1^i^	−19.95 (15)	C6—C5—C4—C3	176.9 (3)
N2—Zn1—S1—O1^i^	160.05 (15)	C1—C2—C3—C4	0.1 (5)
N1—Zn1—S1—O1^i^	80.33 (14)	C12—C4—C3—C2	0.6 (5)
N1^i^—Zn1—S1—O1^i^	−99.67 (14)	C5—C4—C3—C2	−177.8 (3)
O1—Zn1—S1—O1^i^	180.0	C4—C5—C6—C7	1.7 (5)
N2^i^—Zn1—S1—O1	160.05 (15)	C11—C7—C6—C5	−0.2 (5)
N2—Zn1—S1—O1	−19.95 (15)	C8—C7—C6—C5	−179.5 (3)
N1—Zn1—S1—O1	−99.67 (14)	C10—C9—C8—C7	−0.1 (5)
N1^i^—Zn1—S1—O1	80.33 (14)	C11—C7—C8—C9	0.4 (4)
O1^i^—Zn1—S1—O1	180.0	C6—C7—C8—C9	179.7 (3)
O2—S1—O1—Zn1	−119.08 (14)	C1—N1—C12—C4	0.2 (4)
O2^i^—S1—O1—Zn1	118.43 (14)	Zn1—N1—C12—C4	177.7 (2)
O1^i^—S1—O1—Zn1	0.0	C1—N1—C12—C11	178.3 (3)
N2^i^—Zn1—O1—S1	−42.3 (3)	Zn1—N1—C12—C11	−4.2 (3)
N2—Zn1—O1—S1	164.20 (12)	C3—C4—C12—N1	−0.8 (4)
N1—Zn1—O1—S1	85.42 (13)	C5—C4—C12—N1	177.7 (3)
N1^i^—Zn1—O1—S1	−101.66 (13)	C3—C4—C12—C11	−178.9 (3)
O1^i^—Zn1—O1—S1	0.0	C5—C4—C12—C11	−0.4 (4)
N2^i^—Zn1—N1—C1	76.3 (3)	C11—N2—C10—C9	0.2 (5)
N2—Zn1—N1—C1	−179.1 (3)	Zn1—N2—C10—C9	−179.0 (2)
N1^i^—Zn1—N1—C1	127.8 (3)	C8—C9—C10—N2	−0.2 (5)
O1—Zn1—N1—C1	−85.1 (3)	C10—N2—C11—C7	0.2 (4)
O1^i^—Zn1—N1—C1	−19.9 (3)	Zn1—N2—C11—C7	179.5 (2)
S1—Zn1—N1—C1	−52.2 (3)	C10—N2—C11—C12	−178.0 (3)
N2^i^—Zn1—N1—C12	−100.9 (2)	Zn1—N2—C11—C12	1.3 (3)
N2—Zn1—N1—C12	3.66 (19)	C8—C7—C11—N2	−0.5 (4)
N1^i^—Zn1—N1—C12	−49.37 (19)	C6—C7—C11—N2	−179.8 (3)
O1—Zn1—N1—C12	97.7 (2)	C8—C7—C11—C12	177.7 (3)
O1^i^—Zn1—N1—C12	162.9 (2)	C6—C7—C11—C12	−1.7 (4)
S1—Zn1—N1—C12	130.63 (19)	N1—C12—C11—N2	2.0 (4)
N2^i^—Zn1—N2—C10	−92.4 (3)	C4—C12—C11—N2	−179.8 (3)
N1—Zn1—N2—C10	176.6 (3)	N1—C12—C11—C7	−176.3 (2)
N1^i^—Zn1—N2—C10	−13.9 (3)	C4—C12—C11—C7	1.9 (4)
O1—Zn1—N2—C10	76.9 (3)	C13^i^—C14—C13—O3	65.1 (3)

Symmetry codes: (i) −*x*, *y*, −*z*+1/2.

### Hydrogen-bond geometry (Å, °)

**Table:**

*D*—H···*A*	*D*—H	H···*A*	*D*···*A*	*D*—H···*A*
O3—H3B···O2	0.82	1.95	2.727 (4)	157
